# Googling concussion care in the USA: a critical appraisal of online concussion healthcare providers

**DOI:** 10.2217/cnc-2016-0027

**Published:** 2017-03-03

**Authors:** Michael J Ellis, Lesley Ritchie, Erin Selci, Stephanie Grossi, Samantha Frost, Patrick J McDonald, Kelly Russell

**Affiliations:** 1 Department of Surgery, University of Manitoba, Winnipeg, Manitoba, Canada; 2 Department of Pediatrics & Child Health, University of Manitoba, Winnipeg, Manitoba, Canada; 3 Section of Neurosurgery, University of Manitoba, Winnipeg, Manitoba, Canada; 4 Pan Am Concussion Program, Winnipeg, Manitoba, Canada; 5 Children Hospital Research Institute of Manitoba, Canada North Concussion Network, Winnipeg, Manitoba, Canada; 6 Canada North Concussion Network, Winnipeg, Manitoba, Canada; 7 Department of Clinical Health Psychology, University of Manitoba, Winnipeg, Manitoba, Canada; 8 Division of Neurosurgery, British Columbia Children's Hospital, British Columbia, Canada; 9 National Core for Neuroethics, University of British Columbia, Vancouver, British Columbia, Canada

**Keywords:** concussion, healthcare provider, USA

## Abstract

**Aim:**

To examine the online personnel and practice profiles of concussion healthcare providers in the USA.

**Methods:**

We conducted independent, blinded, Google Internet searches for concussion healthcare providers using the terms ‘concussion clinic’ and ‘concussion program’ and each American state and completed a critical appraisal of healthcare personnel and services at these websites.

**Results:**

A total of 184 concussion healthcare providers were identified. Despite offering care to traumatic brain injury (TBI) patients, access to professionals with expertise in TBI including neuropsychologists (40.8%), neurologists (33.7%) and neurosurgeons (21.7%) was variable across sites.

**Conclusion:**

Concussion healthcare in the USA is presently delivered by a range of healthcare professionals with varying levels of training in TBI offering a variety of services.

Concussion is an important public health concern in the USA with 1.6–3.8 million Americans sustaining sports-related concussions alone annually [1]. Although most concussion patients will recover within 1–4 weeks with conservative management, an important subset will present with or experience more complex manifestations of TBI that can include seizures, abnormal neuroimaging findings, vestibulo-ocular dysfunction, exercise intolerance, migraine headaches, postinjury psychiatric disorders or prolonged cognitive impairment [2–4].

In the USA, the acute care and rehabilitation of patients with more severe forms of TBI is largely provided by multi-disciplinary teams of experts in emergency medicine, neurosurgery, critical care and rehabilitation medicine. Within these teams each professional operates within their licensed scope of practice with some aspects of care directed by evidence-based practice guidelines [5]. In contrast, management of patients with concussion and mild TBI in the USA is unregulated. There are also limited national or international guidelines that address the qualified roles and responsibilities of healthcare professionals who offer care to this vulnerable patient population. Consequently, there is public concern that some concussion clinics in North America are operated by professionals with insufficient training in TBI. These clinics may also offer healthcare services they are not optimally trained to provide or services that lack supportive empirical evidence [6,7].

A previous study found wide variability in the personnel and practices of Canadian concussion healthcare providers and clinics as advertised online, that in many cases did not conform to expert consensus guidelines [8]. However, no study has examined the landscape of advertised concussion healthcare in the USA. Accordingly, we conducted online Internet searches to identify advertised concussion healthcare providers in each American state and the District of Columbia, and performed a critical appraisal of the personnel and practices of these providers.

## Methods

Similar to our previous study [8], the Internet search methodology used in this study was developed to simulate searches that could be reasonably carried out by patients or parents seeking to obtain concussion care in the USA. Because this study utilized information that was displayed on publically accessible websites, institutional ethics approval was not required.

### Definitions

For the purposes of this study, we defined concussion healthcare provider as a facility that advertised one or more concussion-related healthcare services on their website. A multidisciplinary concussion healthcare provider was defined as a facility that offered concussion-related services and access to more than one type of healthcare professional (i.e., a sports medicine clinic with access to a sports medicine physician and an athletic therapist) excluding support staff such as physician assistants and nurses. An independent healthcare provider was defined as a healthcare provider or facility that offered access to one type of healthcare provider (i.e., an independent neuropsychologist or a group of sports medicine physicians).

### Stage one: review of concussion healthcare providers

Two authors independently conducted Google searches using the terms ‘concussion clinic’ and ‘concussion program’ in each of the 50 American states as well as the District of Columbia (for example: ‘concussion program’ and ‘Michigan’ and ‘concussion clinic’ and ‘Michigan’). We chose to use Google for this study because it is the most commonly used search engine worldwide [9]. For each individual search, the authors recorded the first three concussion healthcare providers that were identified within the first ten pages of results. All Internet searches were completed on two research computers. Search histories and caches were cleared on each computer prior to initiating each Google search in order to avoid previous searches influencing Google's search algorithms.

Following the independent searches, the two independent authors combined their lists for each state and removed duplicated sites identified with ‘concussion clinic’ and ‘concussion program’ searches to generate a final list of none up to six concussion healthcare providers per state.

### Stage two: critical appraisal of concussion healthcare provider personnel & services

Once the concussion healthcare providers were identified for each state, a standardized data collection form was used to complete a critical appraisal of healthcare personnel and services that were advertised on the providers’ website. Two authors extracted the data with any discrepancies discussed and reviewed by independent moderators. For simplicity, physicians were classified based on their highest level of training. For instance, a pediatrician who indicated specialized training in sports medicine was classified as a sports medicine physician. If a medical doctor (MD) or doctor of osteopathy (DO) was identified but their area of specialization was not indicated, they were classified as a MD or DO without a specified specialty.

The initial Internet searches for concussion healthcare providers were completed on 14 November 2016. Follow-up Internet searches to complete the critical appraisal of personnel and services were completed during the period of 14 November 2016 to 18 November 2016. The results were tabulated and presented as proportions.

## Results

Using the described search methodology, we identified 184 online concussion healthcare providers in the USA, including 141 (76.6%) multidisciplinary and 43 (23.4%) independent concussion healthcare providers. The mean number of concussion healthcare providers identified per state was 3.8 (SD: 0.9; range: 1–6). A summary of identified concussion healthcare providers and personnel is provided in Table 1. A summary of concussion-related services offered by these concussion healthcare providers is provided in Table 2. A wide range of diagnostic and therapeutic services were advertised by the identified concussion healthcare providers. We grouped these services into the following categories: advanced vision, oculomotor, vestibular or balance testing, nutritional supplementation/counseling, cognitive rehabilitation, chiropractic adjustments, neurophysiological testing (e.g., electroencephalography and electromyography), speech therapy, biofeedback/neurofeedback, nerve blocks, conventional neuroimaging studies (e.g., computerized tomography and MRI), advanced neuroimaging studies (e.g., diffusion tensor imaging) and others.

**Table 1.** **Summary of online concussion healthcare providers and personnel in the USA (n = 184).**

**Variable**	**n (%)**

Provider type:	
– Multidisciplinary provider	141 (76.6)
– Independent provider	43 (23.4)

Onsite medical doctor or doctor of osteopathy	151 (82.1)

**Team members**	

Medical professional:	
– Sport medicine physician	91 (49.5)
– Neurologist	62 (33.7)
– Orthopedic surgeon	49 (26.6)
– Rehabilitation medicine physician	54 (29.3)
– Neurosurgeon	40 (21.7)
– Pediatrician	21 (11.4)
– Doctor of osteopathy or physician without a specified specialty	21 (11.4)
– Family medicine physician	19 (10.3)
– Radiologist	14 (7.6)
– Emergency medicine physician	12 (6.5)
– Psychiatrist	11 (6.0)
– Neuro-ophthalmologist/ophthalmologist	8 (4.3)
– Otolaryngologist	5 (2.7)
– Anesthesiologist	2 (1.1)
– Trauma surgeon	2 (1.1)
– Sleep medicine physician	2 (1.1)
– General surgeon	1 (0.5)
– Internal medicine physician	1 (0.5)

Allied health professional:	
– Physiotherapist	78 (43.3)
– Neuropsychologist	75 (40.8)
– Athletic therapist	43 (23.4)
– Occupational therapist	25 (13.6)
– Psychologist	23 (12.5)
– Speech language pathologist	21 (11.4)
– Nurse/nurse practitioner	19 (10.3)
– Physician assistant	10 (5.4)
– Chiropractor	7 (3.8)
– Functional neurologist	7 (3.8)
– Exercise physiologist	4 (2.2)
– Massage therapist	4 (2.2)
– Optometrist	4 (2.2)
– Nutritionist	3 (1.6)
– Dietician	3 (1.6)
– Social worker	2 (1.1)
– Psychometrist	1 (0.5)
– Audiologist	1 (0.5)
– Biomedical engineer	1 (0.5)

**Table 2.** **Summary of concussion-related services offered by concussion healthcare providers.**

**Service (n = 184 providers)**	**Providers, n (%)**
Physiotherapy	76 (41.3)

Baseline testing:	91 (49.5)
– Tool specified	77 (41.8)
– ImPACT	75 (40.8)

Neuropsychological or neurocognitive testing	125 (70.0)

**Alternative diagnostics & therapeutic services**	

Advanced vision, oculomotor, vestibular or balance testing	23 (12.5)

Conventional neuroimaging studies (CT, MRI)	17 (9.2)

Occupational therapy	15 (8.2)

Speech therapy	11 (6.0)

Neurophysiological testing (EMG, NCS)	7 (3.8)

Cognitive rehabilitation	7 (3.8)

Biofeedback/neurofeedback	5 (2.7)

Botulinum toxin injections	4 (2.2)

Advanced neuroimaging studies (DTI, fMRI)	4 (2.2)

Nutritional supplementation/counseling	3 (1.6)

Nerve blocks	3 (1.6)

Massage therapy	3 (1.6)

Vision therapy	3 (1.6)

Chiropractic adjustments	3 (1.6)

Light therapy	2 (1.1)

Sleep testing	2 (1.1)

Trigger point injections	2 (1.1)

Acupuncture	1 (0.5)

TCD ultrasound	1 (0.5)

Dual neurostimulation	1 (0.5)

Audiology	1 (0.5)

Vibration platform therapy	1 (0.5)

Somatosensory stimulation	1 (0.5)

Stool and salivary analysis (brain gut axis and metabolic testing)	1 (0.5)

Soft tissue treatment	1 (0.5)

Soft laser therapy	1 (0.5)

Neuromuscular rehabilitation	1 (0.5)

Eye movement desensitization and reprocessing therapy	1 (0.5)

Cervical epidural injections	1 (0.5)

Cervical facet radiofrequency treatment	1 (0.5)

Craniosacral therapy	1 (0.5)

Facet injections	1 (0.5)

Hemi-stim therapy	1 (0.5)

Low level laser therapy	1 (0.5)

Hyperbaric oxygen therapy	1 (0.5)

CT: Computerized tomography; DTI: Diffusion tensor imaging; EMG: Electromyography; fMRI: Functional MRI; ImPACT: Immediate Post-Concussion Assessment and Cognitive Testing; NCS: Nerve conduction studies; TCD: Transcranial doppler.

## Discussion

This study offers a unique snapshot of the characteristics, personnel and practices of healthcare providers in the USA who advertised concussion-related services on the Internet at the time of our searches. Similar to our previous study of Canadian healthcare providers with websites [8], a number of key findings were identified.

First, the results of this study suggest that there are many specialized concussion clinics in the USA that are operated by a wide range of healthcare professionals with varying levels of training in TBI. In general, the medical diagnosis of concussion or mild TBI is critically dependent on a healthcare provider's trained ability to clinically exclude more serious forms of TBI, associated cervical spine injury, and medical and neurological conditions that can present with concussion-like symptoms or coexist at the time of injury [3]. Accordingly, expert consensus statements conclude that the initial medical evaluation of concussion patients should include a comprehensive clinical history, physical examination and consideration of neuroimaging studies [10]. For patients with persistent symptoms (>10 days), some authors suggest that these assessments be ideally carried out by an experienced sports medicine physician, neurologist or neurosurgeon [3,11]. In the present study, 82.1% of concussion healthcare providers advertised access to an on-site MD (MD or DO). Sports medicine physicians who have clinical training in sports-related concussion were the most common type of physician identified across providers (49.5%). In comparison, access to neurologists (33.7%) who have specialized training in headaches and seizures was more limited as was access to rehabilitation medicine physicians (29.3%) and neurosurgeons (21.7%) who have expertise in the evaluation and management of TBI patients across the full spectrum of severities and etiologies. Among the providers that did not advertise on-site access to a MD, the most common providers identified at these sites were physiotherapists, functional neurologists and neuropsychologists.

In recent years, the clinical management of concussion patients has evolved to incorporate a more multidisciplinary approach. Emerging evidence suggests that patients with prolonged symptoms or postconcussion syndrome can present with clinical features that can be attributed to exercise intolerance, vestibulo-ocular dysfunction, cervical spine injury, migraine headaches, postinjury psychiatric disorders and cognitive impairment that often benefit from targeted rehabilitation approaches [2,3]. These advances have led some experts to recommend that concussion patients with more complex or prolonged symptoms have access to a multidisciplinary team of healthcare professionals who are trained to meet the diverse needs of this unique patient population [2–3,11]. In this study, 76.6% of concussion healthcare providers advertised access to more than one type of healthcare professional at their site including a wide spectrum of subspecialty physicians (e.g., physiatrists, psychiatrists and neuro-ophthalmologists) and allied health professionals (e.g., physiotherapists and occupational therapists). Although these trends would seem to reflect a shift toward a multidisciplinary approach, a more critical evaluation demonstrates that there are few concussion healthcare providers or clinics with access to the complete complement of multidisciplinary professionals required to meet the diverse needs of concussion patients. For instance, among the concussion healthcare providers identified in this study, only 40.0% advertised on-site access to both an MD and a neuropsychologist and only 19.0% advertised access to an MD, neuropsychologist and physiotherapist. Among the concussion healthcare providers that were classified as independent providers and identified on-site healthcare professionals, these sites most commonly offered access to sports medicine physicians, physiotherapists and functional neurologists.

Second, the results of this study suggest that concussion healthcare providers are offering a diverse number of services to concussion patients, some of which are provided by experts with suboptimal training and some that have a limited base of supportive evidence. The most common service provided by concussion healthcare providers identified in this study was neurocognitive and neuropsychological testing. At present, concussion expert consensus opinion suggests that while neuropsychological testing can be helpful in the postinjury management of selected concussion patients, there is insufficient evidence to support the widespread use of baseline neurocognitive testing [10,12]. Furthermore, there is strong expert consensus that neuropsychologists are the only healthcare professionals who possess the optimal training to administer and interpret neurocognitive and neuropsychological testing in patients with concussion [12,13]. In some cases, psychologists may also be familiar with the psychometric properties of neuropsychological testing instruments but their experience performing these tests in TBI patients may be variable. In this study, we found 49.5% of concussion healthcare providers offered baseline testing with the vast majority using the Immediate Post-Concussion Assessment and Cognitive Testing instrument. Of those 125 that advertised neurocognitive or neuropsychological testing as a service on their website, only 45.6% listed access to a neuropsychologist or psychologist. These findings are consistent with trends observed among medical teams caring for collegiate athletes in the USA where neuropsychologists are often not involved in baseline and postinjury neurocognitive testing [14]. It is possible that some concussion healthcare providers identified in this study had obtained additional training or certification from private companies that distribute neurocognitive assessment tools. However at present, there does not appear to be a consensus among neuropsychologists in the field as to whether this training is sufficient for non-neuropsychologists to offer this service to concussion patients. Nevertheless, the findings of this study suggest that neuropsychological testing at many sites is being undertaken by healthcare professionals with suboptimal training in the psychometrics, administration and interpretation of neuropsychological testing and that many clinics do not have the professional resources to meet the needs of patients with persistent cognitive impairments following concussion.

In recent years, emerging research has begun to establish a role for active rehabilitation strategies in patients with persistent concussion symptoms [2,3]. One previous randomized controlled trial demonstrated enhanced recovery among sports-related concussion patients who received cervicovestibular physiotherapy compared with those who did not [15]. There are also accumulating studies that suggest that concussion and mild TBI patients with persistent symptoms may also benefit from tailored submaximal aerobic exercise prescription [16–19]. Together, these advances may reflect the number of concussion healthcare providers that offer access to physiotherapists (43.3%), athletic therapists (23.4%) and occupational therapists (13.6%). In addition to these services, there were providers who offered access to potentially costly alternative diagnostic and therapeutic services, some that have limited to no empirical evidence to support their use in concussion patients. For example, some providers offered access to advanced neuroimaging studies including diffusion tensor imaging, functional neurocognitive imaging and positron emission tomography even though experts agree that techniques such as these should be limited to research only [10]. Other providers offered access to hyperbaric oxygen therapy, neurofeedback, chiropractic adjustments and light therapy interventions for which there is no firm evidence to support a therapeutic benefit in concussion patients.

Although there was wide variability in the advertised personnel and practices among American concussion healthcare providers, the results of this study suggest a notably higher standard of concussion healthcare in the USA compared with Canada. A previous study found that only 40% of Canadian concussion healthcare providers advertised access to an on-site MD with many clinics appearing to be operated solely by chiropractors and physiotherapists. Access to healthcare professionals with expertise in TBI, such as, neuropsychologists (28%), neurologists (7%) or neurosurgeons (5%), was limited across Canadian concussion healthcare providers. 67% of concussion healthcare providers advertised baseline testing on their website. Among those that advertised baseline Immediate Post-Concussion Assessment and Cognitive Testing neurocognitive testing on their websites, only 21% identified a neuropsychologist as a member of the clinical team [8].

The present study has several limitations. First, it is possible that certain concussion healthcare providers were not identified using our search methodology. This would have occurred if the provider did not have a website. Alternatively, some providers may have engaged in online marketing strategies that altered where their website appeared in the results for online searches using keywords such as ‘concussion clinic’. Second, the critical appraisal of concussion healthcare provider personnel and practices was based solely on information gathered by the authors from the provider's website. In many cases, the healthcare providers and their designated areas of specialization were well documented on the providers’ website, while in other cases it was unclear which providers were specifically assigned to a concussion clinic operating within a much larger medical facility. Therefore, it is possible that certain concussion healthcare providers and clinics had access to additional healthcare personnel or offered services that were not captured by our searches. In contrast, it is also possible that certain concussion healthcare providers advertised access to professionals with licensed training in certain aspects of TBI management (i.e., neurosurgeons, neurologists and rehabilitation medicine physicians) on their website, but that these individuals do not participate in the day-to-day clinical care of concussion patients at these facilities. In addition, some providers may have established collaborations with outside specialists who were not identified on their websites. Acknowledging these limitations, these study findings should not be viewed as a concise summary of concussion healthcare services across the USA but should instead be viewed as a representative snapshot of online information available to concussion healthcare consumers at the time of this study.

## Conclusion

In summary, the results of this study suggest that specialized concussion and mild TBI healthcare in the USA is provided by a wide range of healthcare professionals with varying levels of clinical training and experience in TBI. While some providers and clinics offered access to a multidisciplinary team of medical and allied healthcare professionals with expertise across the full spectrum of TBI, others offered access to a limited number of healthcare professionals who may not be fully qualified or equipped to meet the diverse needs of concussion patients. Future studies are needed to examine potential barriers to involve certain healthcare providers, in particular neuropsychologists, in the operation of specialized concussion clinics. Overall, the results of this study point to an urgent need for medical experts in this field to clearly define the qualified roles and responsibilities of healthcare professionals who participate in the care of this TBI population. Despite these limitations, the standard of concussion healthcare in the USA appears to be notably higher than that available in Canada.

**Executive summary**

- Concussion healthcare is presently unregulated in the USA and no previous study has examined the personnel and practices of online advertised concussion healthcare providers in this country.
- We conducted a critical appraisal of healthcare personnel and services among concussion healthcare providers identified by Google internet searches for the terms ‘concussion clinic’ and ‘concussion program’ and each American state.
- Among the 184 healthcare providers that offered medical services to this traumatic brain injury population, access to healthcare professionals with clinical expertise in traumatic brain injury including neuropsychologists, neurologists or neurosurgeons was variable.
- The results of this study suggest that national and international guidelines addressing the requisite training and qualifications as well as the roles and responsibilities of healthcare professionals participating in the multidisciplinary care of concussion patients are urgently needed.
